# Supplementary material for: Infrared laser-induced gene expression for tracking development and function of single C. elegans embryonic neurons
Source: Nat Commun. 2017 Jan 18;8:14100. doi: 10.1038/ncomms14100 (PMC5253673; doi:10.1038/ncomms14100)
Supplement: Supplementary Information — Supplementary Figures, Supplementary Tables and Supplementary References. [file ncomms14100-s1.pdf]

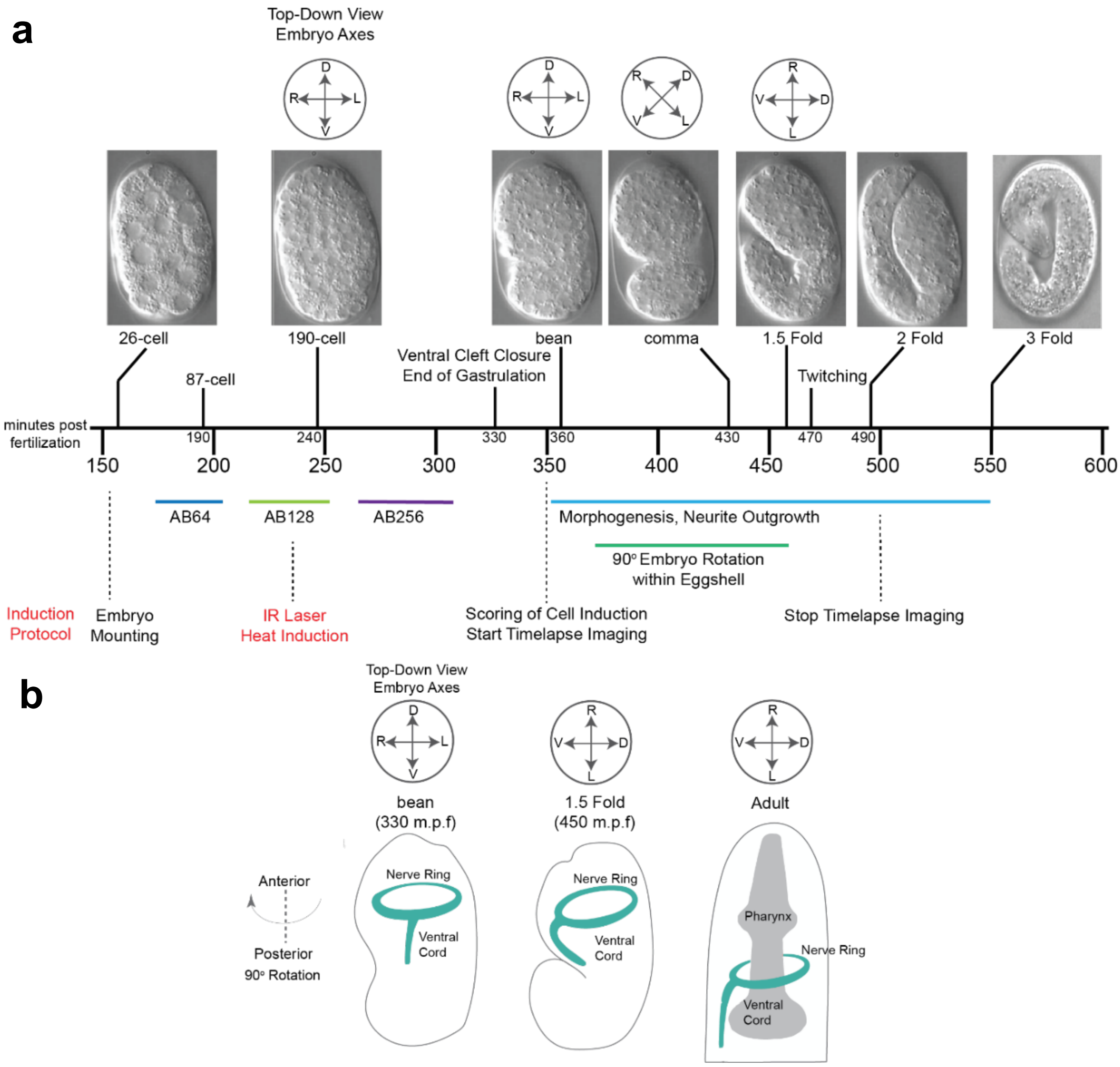

Supplementary Figure 1. Time Line of *C. elegans* Embryonic Development.

(a) Proliferation occurs from 0-360 min post fertilization (m.p.f.) and morphogenesis begins at the bean stage (360 m.p.f.), when the major organs form and neurite outgrowth begins. Most neurons are derived from the AB lineage. The stage ABn corresponds to the stage when the AB precursor cell has generated *n* progeny cells. IR induction protocol is shown below time line. Embryos are dissected from hermaphrodites (<150 m.p.f.) and mounted. The AB128 stage is used for identification of precursor cells using nuclear markers and for inducing cells by IR irradiation. Most cells at the AB128 stage will divide twice to generate four progeny cells. By the

bean stage, most cell divisions are complete, and induction of fluorescent reporters is visible. Time-lapse imaging is initiated to visualize neurite outgrowth. Twitching of the embryo begins at 470 m.p.f.

(b) Placement of the nerve ring and ventral cord is shown for the bean and 1.5-fold stages for comparison to embryonic imaging experiments presented in the paper. Under slight compression, the embryo undergoes stereotyped movements, rotating 90° within the eggshell soon after the onset of morphogenesis, turning from the dorsal-ventral axis to the left-right body axis aligning to the Z-imaging axis.

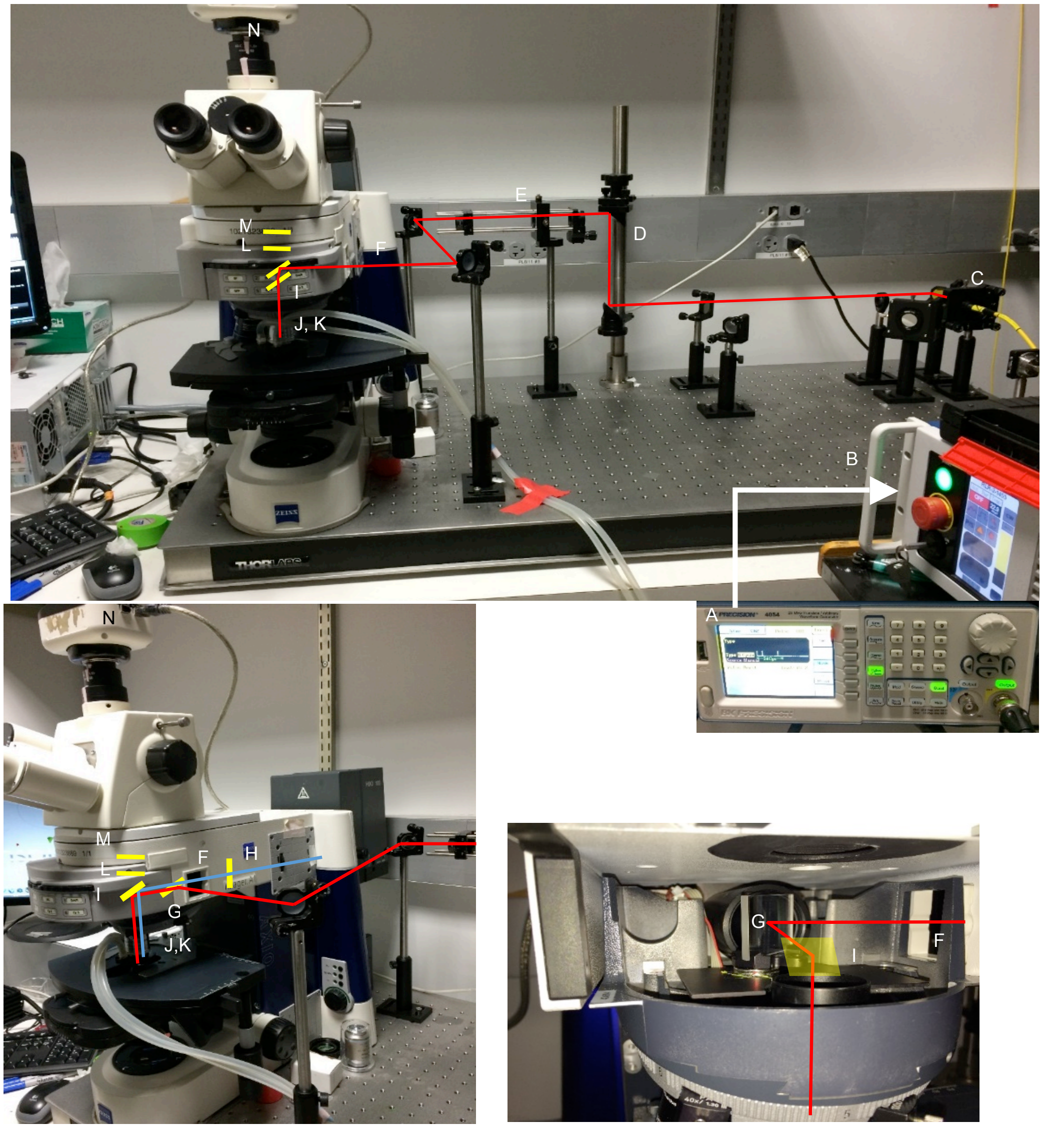

A: Signal Generator
B: 1455 nm Single-Mode Laser
C: Fiber Head with Collimator
D: Periscope
E: Beam Expander (f_1 = 35 mm, f_2 = 200 mm)
F: Microscope Port to Infinity Space
G: Short-Pass Dichroic
H. Dual Excitation Filter
I. Dual Dichroic
J: 100x, 1.3 N.A. Objective
K: Objective Cooling Ring
L: Emission Filter
M: IR Protective Filter
N: CCD Camera

Supplementary Figure 2. Optical Design and Construction. The power and duration of the infrared laser pulse is controlled by a signal generator (A) connected to the external interface of the laser (B). After collimation (C), the beam is expanded by two lenses (E) and then directed to the side port of the microsope (F, Zeiss AxioImager A1), accessing the infinity space before the

imaging dichroic. Here, we placed a shortpass dichroic (made removable by affixing a circular magnet to the base of the dichroic mount and another magnet to the microscope itself) to merge the excitation beam (blue) and the IR beam (red). The dual dichroic (I) and emission filter (L) for imaging mCherry/GFP and reflecting IR are placed in the filter turret. An additional IR filter (M) is placed before the camera and eyepieces to block any residual IR radiation. A temperable ring (K) is affixed to the objective (J) and connected by tubing to a temperature-controlled water bath (not shown) to cool the sample. Part specifications can be found in Methods.

Supplementary Figure 3

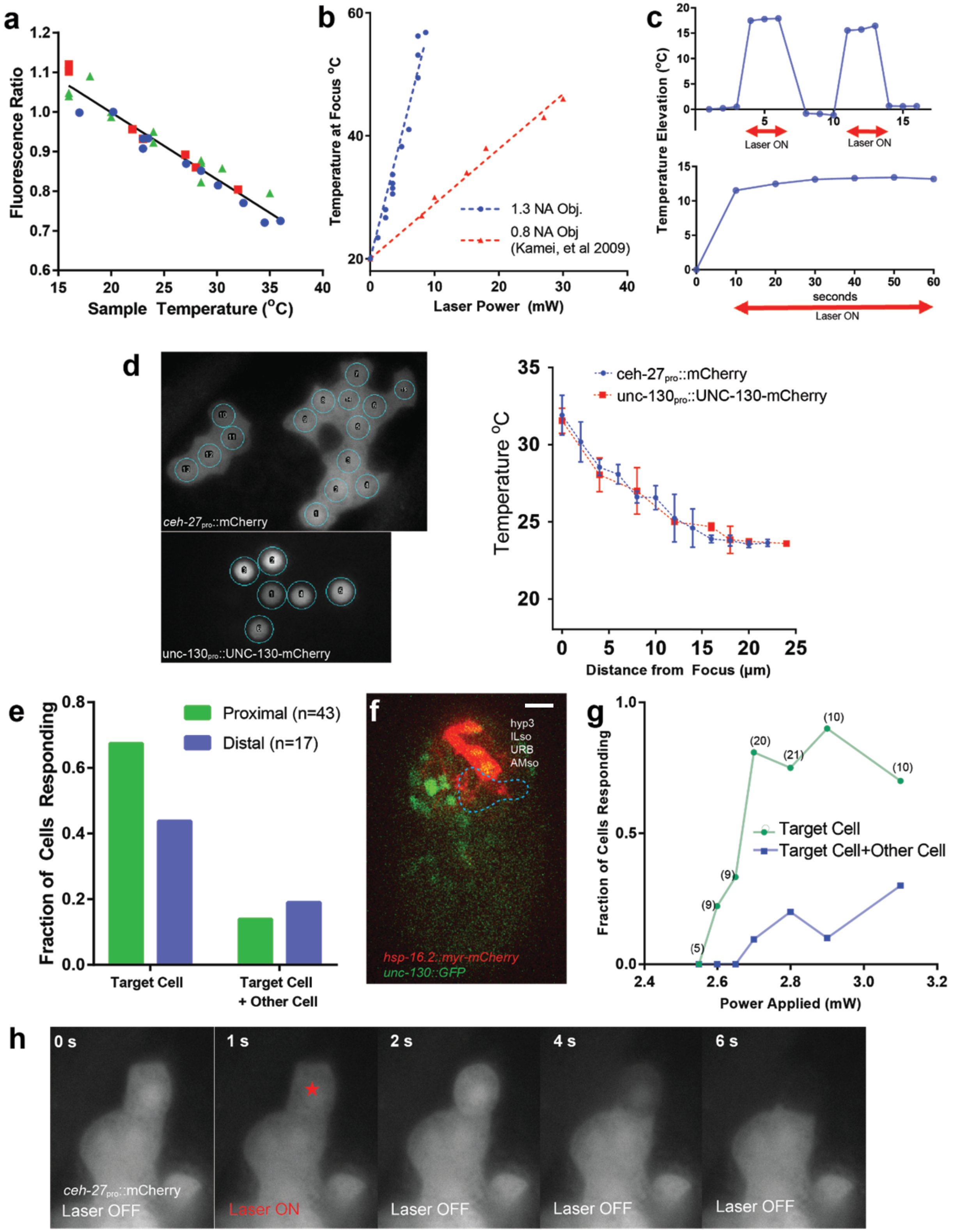

Supplementary Figure 3. Temperature Calibration of IR Induction Gene Setup.

(a) Temperature dependence of *mCherry* in vivo. Three independent samples are represented by different colors. For some temperatures, multiple values were taken after temperature transitions to confirm reversibility.

(b) Comparison of laser power applied vs. temperature at focus for 1.3 NA objective and 0.8 NA objective. In a previous setup[1], a 1.3 NA objective was used for larval induction experiments, as in our embryonic studies, but a 0.8 NA objective was used for temperature measurements (red curve, taken from [1]). The authors suggest that the temperature reached during induction experiments with the higher 1.3 NA objective is the same as what is measured. Our i*n vivo* temperature measurement with a 1.3 NA objective (blue curve) shows that the temperature vs power curve is much steeper than previously described. Thus, the Kamei et al. induction conditions (11 mW, 1 second) elevate the temperature to 65°C, causing extensive cell damage in the embryo. The optimized method we present, which raises cells to much lower temperatures (3 mW, reaching ~32°C, see Fig. 1e-f), achieves high gene induction frequency because the much longer duration of heat stress (5 minutes vs. 1 second, see also Supplementary Fig. 1c) compensates for the lower temperature, resulting in a strong heat shock response.

(c) Temperature reaches steady state immediately during and after IR laser Induction (top). The laser (4.5 mW) is pulsed between on and off states every 3 seconds, with the camera exposure synchronized to the laser-on time. Note that the temperature change occurs within the first frame and then returns to baseline after each pulse. Bottom, heat does not accumulate at the focus over long time periods. The laser (3.5 mW) is turned on at t=10 s, coinciding with the camera exposure, and images are acquired every 10 seconds.

(d) Comparison of *in vivo* temperature measurement using cytoplasmic and nuclear mCherry markers. Subtle cell shape changes and dispersion of tissue occur during laser irradiation of cells. To confirm that fluorescence decrease was due to temperature changes rather than dilution of fluorescent protein, a nuclear mCherry marker ($\textit{unc-130}_{\mathrm{pro}}$::UNC-130-mCherry) was used for comparison. As the entire fluorescence from a cell can be measured in these experiments, without concern for the actual boundaries drawn (below), the summed value should be insensitive to any changes in cell shape or movement. No differences were observed in the measured temperature or distribution using different markers.

(e) Fluorescence induction is only weakly affected by cell location. Due to embryo compression during mounting, the dorsal-ventral axis of the embryo aligns along the z-axis of imaging. UNC-130-GFP-labeled ABplaapaa or ABplaapap lie on the dorsal surface of the embryo, and are therefore either close to the coverslip (proximal) or close to the slide surface (distal).
(f) Induction of non-target cells. Cells in addition to the target cell are labeled at low frequency (<20%). Here ABplaapaa was targeted, and the progeny, named in white, are labeled brightly by myr-mCherry and co-labeled by UNC-130-GFP nuclear signal. Additional weakly labeled cells indicate off-target induction (blue outline) and are not co-labeled by UNC-130-GFP. Scale bar, 5 μm.
(g) Fluorescence induction plotted against laser power at AB64. Irradiation is performed on the target cell ABplaapa, and specificity is scored using an *unc-130* reporter. Number of embryos irradiated for each condition is indicated.
(h) Disintegration of embryonic cells after induction with previously described conditions[1] (11 mW, 1 second). In frame 2, the laser is turned on, and in frame 3, the laser is turned off. Shortly after, the cell begins to round and the fluorescence leaks into the extracellular space, probably due to a membrane disruption.

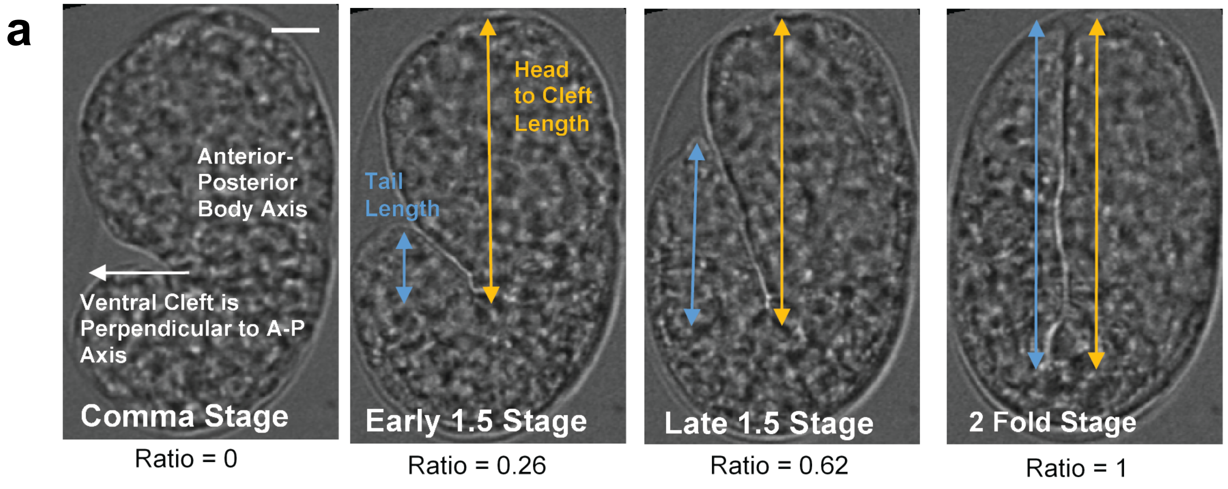

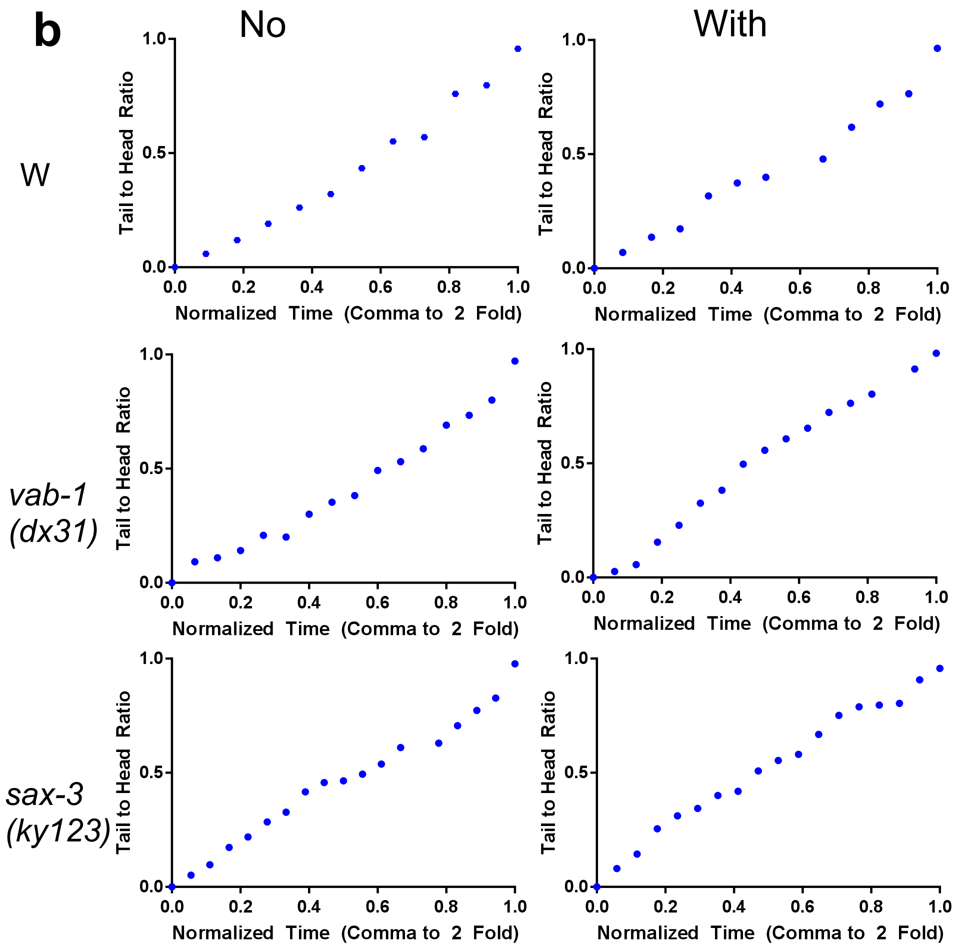

c

Avg, STD (min)

	No Imaging	With Imaging
Wildtype	58.6 (6.83)	70.8 (8.6)
vab-1 (dx31)	73.3 (5.1)	82.1 (13.2)
sax-3 (ky123)	81.6 (11.7)	85.8 (13.9)

Supplementary Figure 4. Tail-to-Head Ratio Is Used as a Proxy for Developmental Stage.

(a) Calculating Tail-to-Head Ratio. Comma stage occurs when the ventral cleft is perpendicular to the anterior-posterior embryo axis [2]. At 2-fold, the tail overlaps entirely with the head.

(b) Tail-to-head ratios plotted against normalized time in imaged and non-imaged, wild-type and mutant embryos. Normalized time calculated by setting comma stage to 0 and 2-fold stage to 1. After twitching (Ratio = 0.5-0.6), the tail occasionally retracts, and values for these are omitted. Without imaging, ratio curves steepen over time. With fluorescence imaging, mutants, unlike wild-type embryos, sometimes show flattening of the curve towards time course end, suggesting phototoxic developmental slowdown. To overcome this, Tail-to-Head Ratio was used to mark developmental time rather than absolute or normalized time.

(c) Average duration (SD) from comma to 2-fold (n=6 embryos/condition).

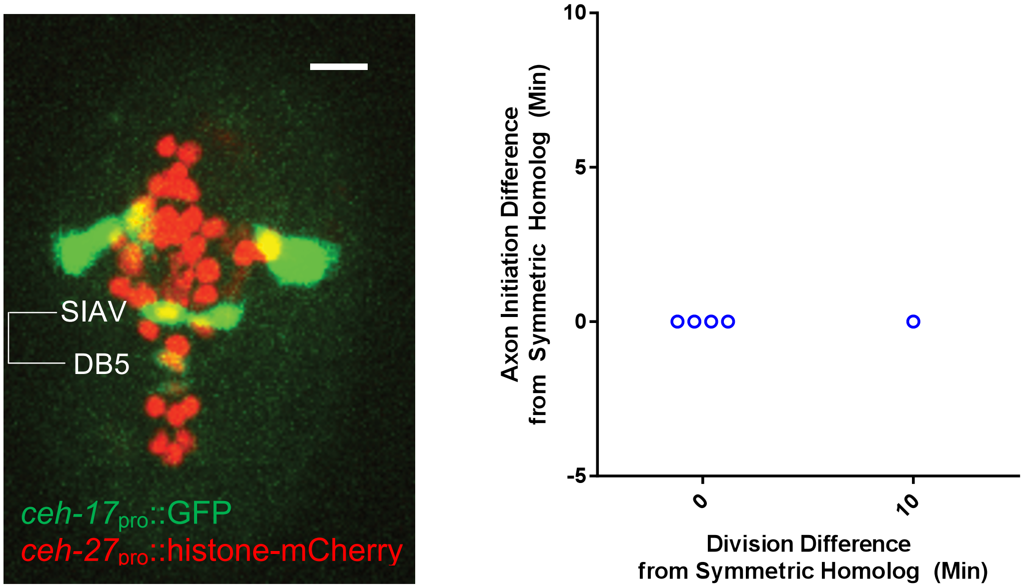

Supplementary Figure 5. Natural Variation in Birth Time Between Left-Right Homologs Does Not Cause Changes in the Timing of Axon Outgrowth. Left, neurons were marked sparsely and symmetrically using *ceh-17*$_{pro}$::GFP, and the precursors of those cells (and others) were marked using *ceh-27*$_{pro}$::histone-mCherry. Scale bar, 5 µm. Right, the SIAV/DB birth time was correlated to the axon outgrowth time of SIAV. Despite changes in the timing of divisions in the left-right precursor cells, axons of the descendants always grow out simultaneously. Images were acquired every 5 minutes.

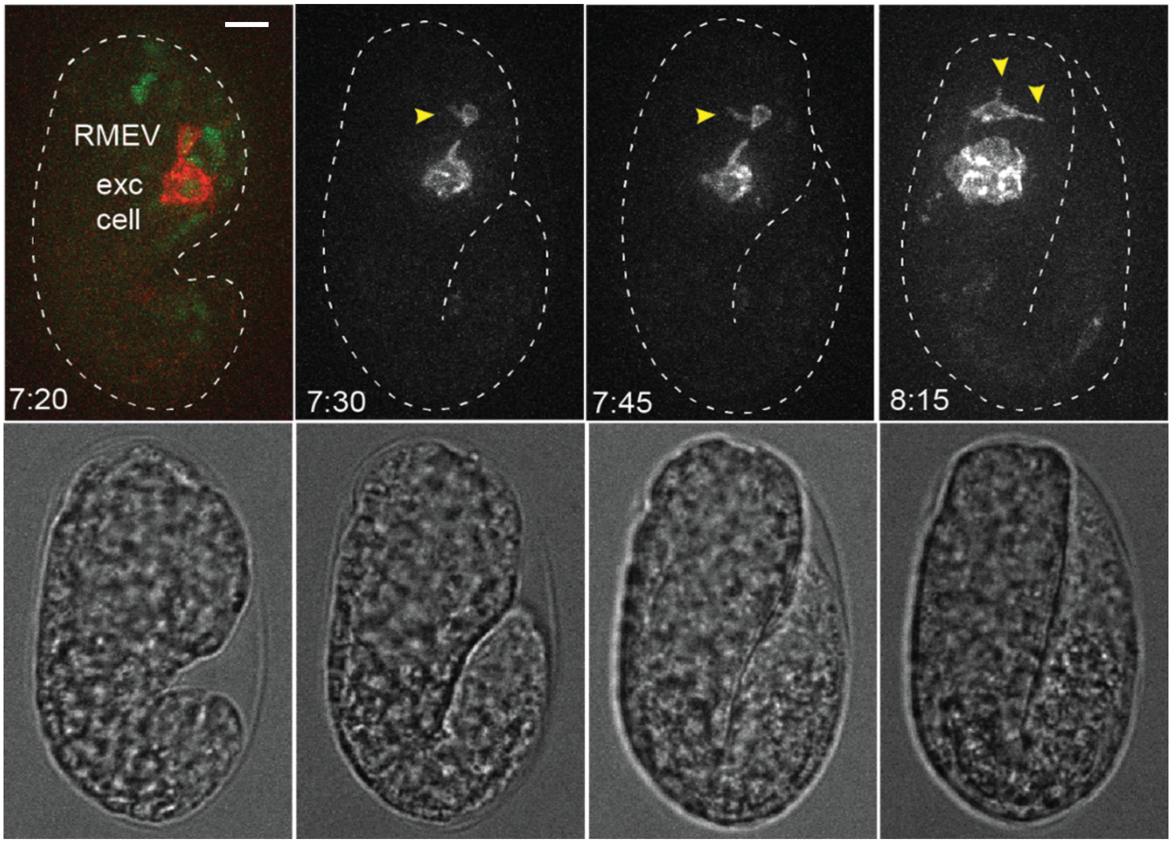

Supplementary Figure 6. RMEV Axon Outgrowth. Axon growth for RMEV initiates at 7h:30m post-fertilization. Axon growth into the nerve ring continues in the 2-fold embryo (8h:15m). Scale bar, 5 μm.

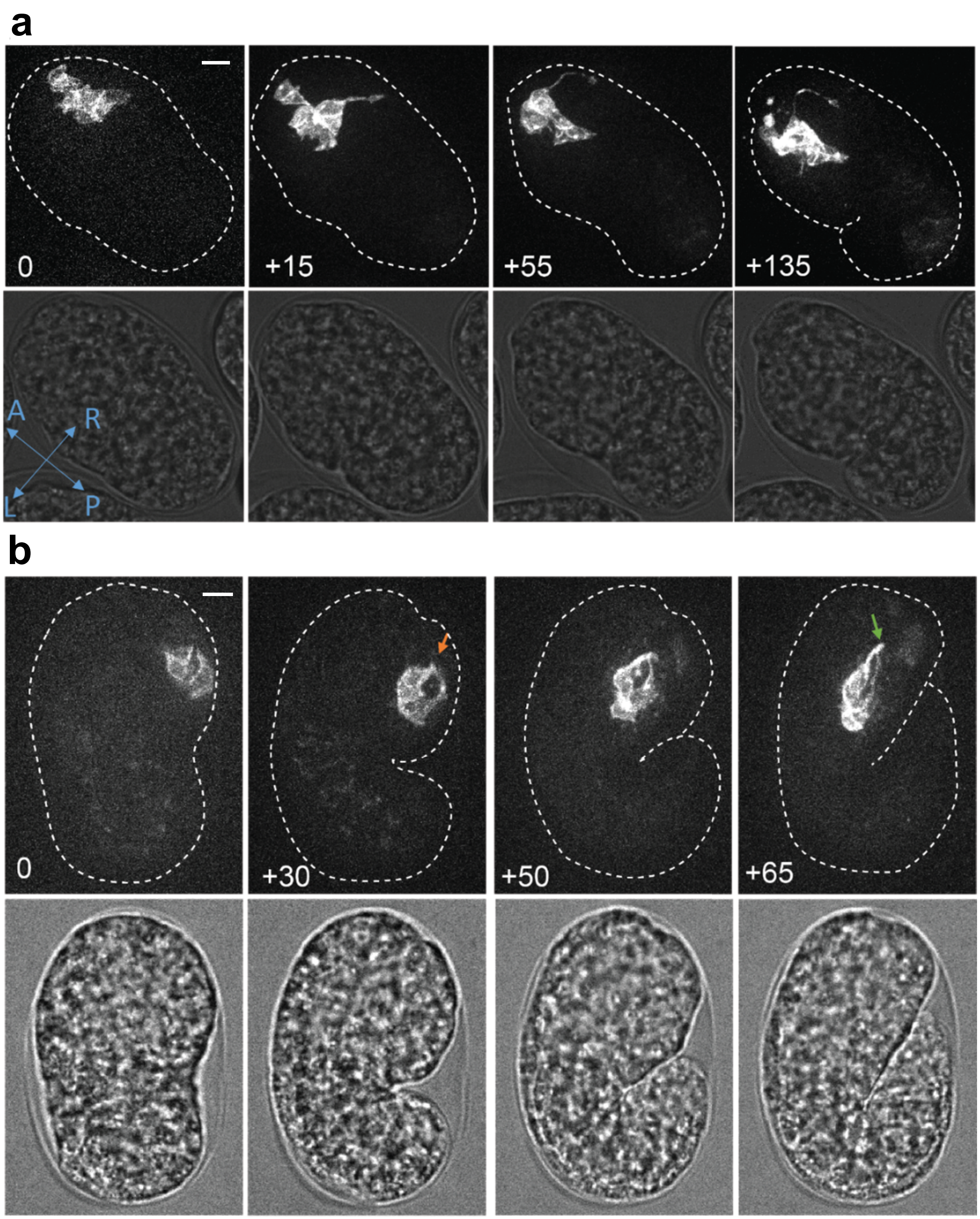

Supplementary Figure 7. Defects in Dendrite Placement in *sax-3(ky123)*.

(a) Dendrite extension of amphid neurons occurs in the anterior-posterior axis in wild-type animals (Figure 3c, Supplementary Movie 1). In one *sax-3(ky123)* embryo, the dendrite begins to extend in the left-right axis before turning and correcting its trajectory to the anterior-posterior axis. This embryo arrested prior to twitching. Scale bar, 5 µm.

(b) Dendrite extension occurs in 2 separate bundles (red arrow), rather than one, as normally seen in wild-type animals. The processes do not extend to the nose-tip in the 2-fold embryo (green arrow), indicating that the dendrites are shorter than normal. Time for both time lapse image series are given in minutes from the first image shown. Scale bar, 5 μm.

Strains and Transgenes

Supplementary Table 1. Transgenes Used in this Study

Transgene	Constructs
nsIs520	pANU01(*hsp-16.2*::Cre recombinase) + pEKL15(*lin-15*(+))
nsIs515	pANU13(*his-72* $_{pro}$:lox-STOP-lox::myr-GFP) + pRF4(*rol-6*).
nsIs420	pANU21(*hsp-16.2*::myr-GFP) + pEKL15(*lin-15*(+))
nsIs427	pANU43(*hsp-16.2*::myr-mCherry) + pEKL15(*lin-15*(+))
wgIs76	*unc-130*::TY1::eGFP::3xFLAG
kyIs37	*odr-10*$_{pro}$::GFP
oyIs45	*odr-1* $_{pro}$::YFP
nsEx5290	pANU84(*ceh-27* $_{pro}$::GFP) + pANU85(*ceh-27* $_{pro}$*::mCherry*) + pEKL15(*lin-15(+)*)
nsEx5291	pANU107(*unc-130* $_{pro}$ (5.5kb)::UNC-130-GFP) + pANU110(*unc-130* $_{pro}$ (5.5kb)::UNC-130-mCherry)
nsEx5292	pEP7(*hsp-16.2*::*daf-6*) + pANU107(*unc-130* $_{pro}$ (5.5kb)::UNC-130-GFP) + pRF4(*rol-6*)
nsEx5065	pANU134(*unc-130* $_{pro}$ (5.5kb)::UNC-130*::*STOP::SL2::Cre) + pANU53(*dyf-7* $_{pro}$:: lox-STOP-lox::myr-GFP) + pEKL15(*lin-15(+)*)

Supplementary Table 2. Mutant Alleles Used in this Study

vab-1(dx31) II
sax-3(ky123) X
daf-6(e1377) X
lin-15(n765ts) X

Supplementary Table 3. Plasmids Constructed/Used in this Study

pANU01	*hsp-16.2*::Cre	*hsp-16.2* was digested from pPD49.78 (gift from A. Fire) and inserted into pEM3 = *ncs-1* $_{pro}$::Cre (gift from C. Bargmann, from[3] using SphI/XmaI sites
pANU21	*hsp-16.2*::myr-GFP	*hsp-16.2* was digested from pPD49.78 (gift from A. Fire) and inserted into pMH29 [4] using SphI/XmaI sites
pANU43	*hsp-16.2*::myr-mCherry	Myristylation sequence MGSCIGK inserted at N-terminus of mCherry [5]<br>myr-mCherry was cloned into pANU21 using AgeI/EcoRI sites
pANU13	*his-72* $_{pro}$::lox-STOP-lox::myr-GFP	A 1kb *his-72* promoter [6] was amplified from genomic DNA using oligos 5' aaccCTGCAGaaacgttatagtgtggacaccaattt and 3' aaccCCCGGGtgttgttctggaaattgagaattga and cloned into pMH29 (Heiman and Shaham, 2009) using PstI/XmaI sites

		loxP- *LacZ*STOP -loxP was amplified from pEM1 using oligos<br>5' aaccCCCGGGcaggaggacccttggctagcgataa<br>3' aaccACCGGTgataacttcgtataatgtatgctat<br>and cloned into vector from above using SmaI/AgeI sites
pANU53	*dyf-7* $_{pro}$::lox-STOP-lox::myr-GFP	lox-STOP-lox was amplified from pEM1(gift of C. Bargmann, from[3] using oligos<br>5' aaccGGATCCcaggaggacccttggctagcgataa<br>3' aaccCCCGGGgataacttcgtataatgtatgctat<br>and cloned into pMH29 (Heiman and Shaham, 2009) using BamHI and XmaI sites
pANU84	*ceh-27*$_{pro}$::GFP	*ceh-27*$_{pro}$(3.3 kb) was amplified using<br>5' aaccCTGCAGttcgtttgttgtttcactttctgagga<br>3' aaccGGATCCtctacaaattaattgtagttaaaggcgagaaactgg
pANU85	*ceh-27* $_{pro}$::mCherry	See pANU84
pANU107	*unc-130*(5.5kb) $_{pro}$:: UNC-130-GFP	*unc-130* $_{pro}$ (5.5)::*unc-130*(CDS) without the last 15 amino acids was amplified from N2 genomic DNA using oligos<br>5' aaccCCTGCAGGaccgatcttgcgattcacttagtg<br>3' aaccCCCGGGagctcgatgaagttctcttctgg<br>cloned in frame into pPD95.75 using SbfI/XmaI
pANU110	*unc-130*(5.5kb) $_{pro}$:: UNC-130-mCherry	see pANU107
pANU134	*unc-130*(5.5kb) $_{pro}$::UNC-130::STOP::SL2::Cre	*unc-130* $_{pro}$ (5.5)::*unc-130*(CDS)::STOP was amplified from N2 genomic DNA using<br>5' aaccCCTGCAGGaccgatcttgcgattcacttagtg<br>3' aaccCCCGGGctagctcgatgaagttctcttctgg<br>SL2-nCre was generated using fusion PCR<br>(used to drive cre expression with regulation of *unc-130* from promoter and introns)
pEP7	*hsp-16.2*::*daf-6*	[7]

Supplementary References

1. Kamei, Y. *et al.* Infrared laser-mediated gene induction in targeted single cells in vivo. *Nat. Methods* **6,** 79–81 (2009).

2. Schnabel, R., Hutter, H., Moerman, D. & Schnabel, H. Assessing normal embryogenesis in Caenorhabditis elegans using a 4D microscope: variability of development and regional specification. *Dev. Biol.* **184,** 234–65 (1997).

3. Macosko, E. Z. *et al.* A hub-and-spoke circuit drives pheromone attraction and social behaviour in C. elegans. *Nature* **458,** 1171–5 (2009).

4. Heiman, M. G. & Shaham, S. DEX-1 and DYF-7 establish sensory dendrite length by anchoring dendritic tips during cell migration. *Cell* **137,** 344–55 (2009).

5. Adler, C. E., Fetter, R. D. & Bargmann, C. I. UNC-6/Netrin induces neuronal asymmetry and defines the site of axon formation. *Nat. Neurosci.* **9,** 511–8 (2006).

6. Murray, J. I., Bao, Z., Boyle, T. J. & Waterston, R. H. The lineaging of fluorescently-labeled Caenorhabditis elegans embryos with StarryNite and AceTree. *Nat Protoc* **1,** 1468–76 (2006).

7. Perens, E. A. & Shaham, S. C. elegans daf-6 encodes a patched-related protein required for lumen formation. *Dev. Cell* **8,** 893–906 (2005).
